# Explantation of aortic infrarenal stent graft

**DOI:** 10.1308/003588412X13373405385214i

**Published:** 2012-07

**Authors:** J Krysa, PR Taylor

**Affiliations:** Guy’s and St Thomas’ NHS Foundation Trust,UK

This technique has helped us to achieve proximal control during stent graft explantation. A large Foley catheter is inserted through a disconnected limb of stent graft and placed in the suprarenal aorta. Inflation of the balloon provides proximal control (Fig 1). The proximal end of the stent graft is extracted from the aorta. If another graft is used, proximal anastomosis is carried out with the balloon inflated. To complete the anastomosis a second foley catheter is passed through a limb of the new graft (Fig 2). while the original catheter is deflated and removed, the second balloon is inflated in the suprarenal aorta (Fig 3) and the anastomosis completed.

**Figure 1:**
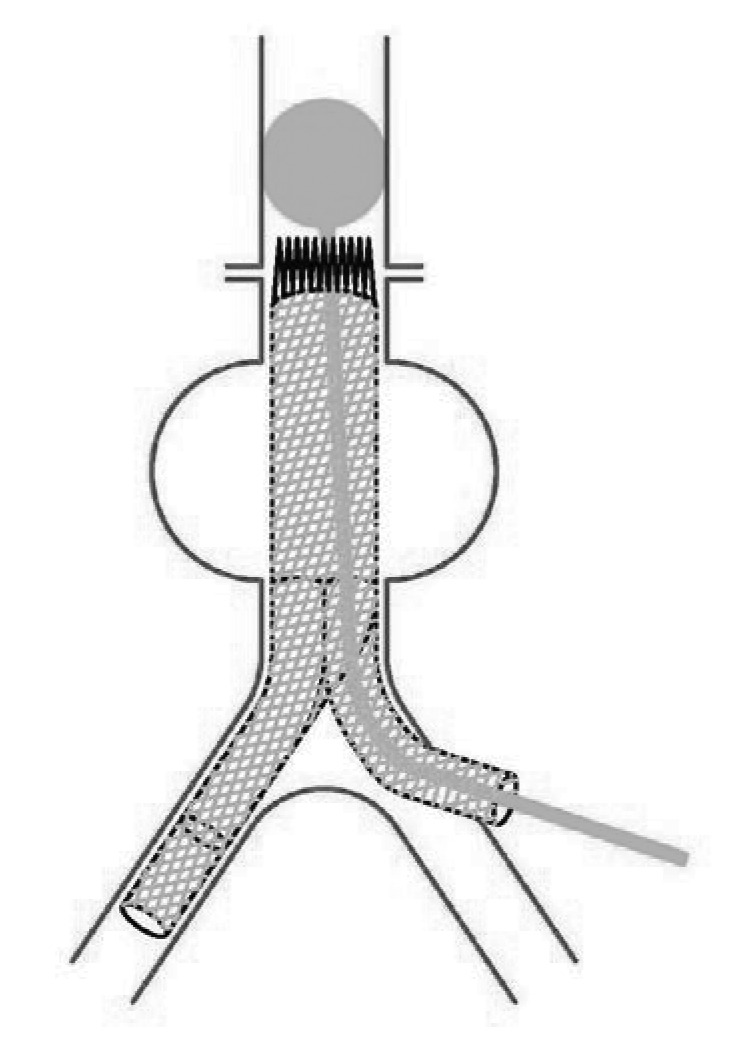
A Foley catheter is inserted through the stent graft and placed in the suprarenal aorta, after which the balloon is inflated.

**Figure 2:**
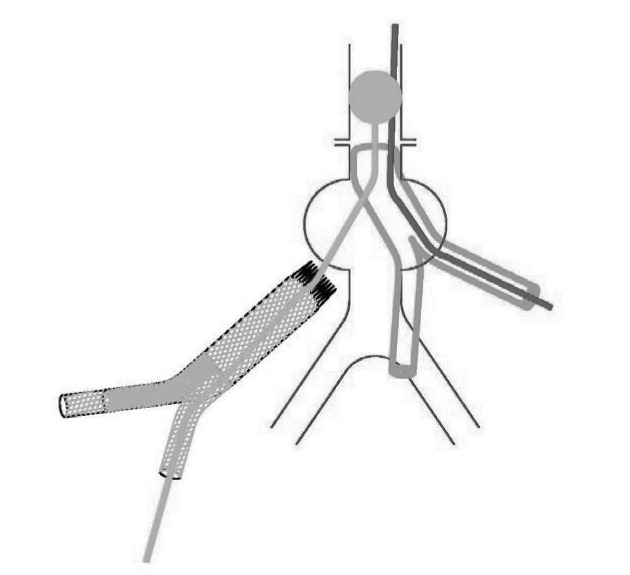
A second foley catheter is passed through a limb of the new graft.

**Figure 3:**
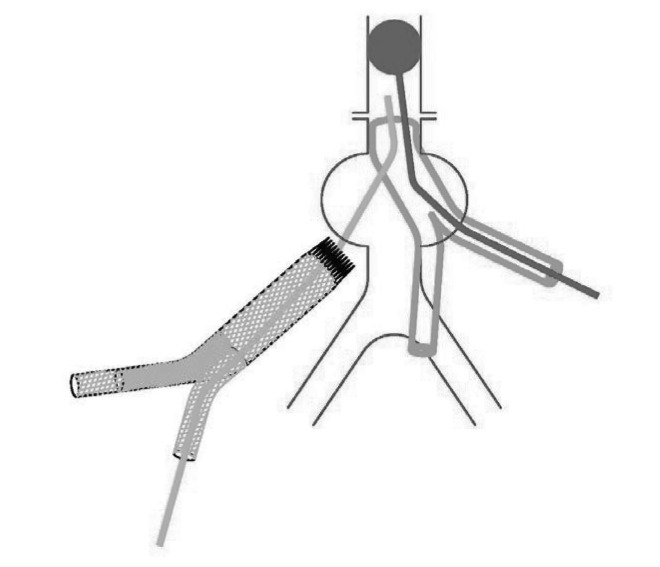
The original catheter is deflated and removed, and the second balloon is inflated in the suprarenal aorta.

## Acknowledgement

We would like to thank James Clark for his help with the drawings.

